# Correlation Between Thyroid Hormone Profiles and Kidney Disease: Improving Global Outcomes (KDIGO) Risk Categories in Diabetic Kidney Disease Patients

**DOI:** 10.7759/cureus.81781

**Published:** 2025-04-06

**Authors:** Kashif Abdullah, Anjum Shahzad, Seemab Javaid, Muhammad Albaz Khan Chandia, Muhammad Jamil, Adnan Ahmad Zafar, Alia Mirghani Ahmed Mirghani, Muhammad Irfan Jamil, Adeel Ahmed

**Affiliations:** 1 Nephrology, Chelsea and Westminster Hospital NHS Foundation Trust, London, GBR; 2 Nephrology, Lahore General Hospital, Lahore, PAK; 3 Nephrology, Rashid Hospital, Dubai, ARE; 4 Nephrology, Sir Ganga Ram Hospital, Lahore, PAK; 5 Internal Medicine, Nishtar Medical University, Multan, PAK; 6 Nephrology, Islam Medical & Dental College, Sialkot, PAK; 7 Pathology, University of Gezira, Wad Madani, SDN; 8 Medicine, Lahore General Hospital, Lahore, PAK

**Keywords:** chronic kidney disease (ckd), diabetic kidney disease (dkd), kidney disease: improving global outcomes (kdigo) classification, thyroid disorder, thyroid-stimulating hormone

## Abstract

Background and aim

Thyroid dysfunction is commonly observed in individuals with diabetic kidney disease (DKD) and may influence disease progression. This study aimed to investigate the relationship between thyroid hormone profiles and Kidney Disease: Improving Global Outcomes (KDIGO) risk stratification in patients with DKD.

Methods

A cross-sectional analysis was conducted on 350 patients diagnosed with DKD, recruited from the nephrology outpatient department at Lahore General Hospital between December 2023 and June 2024. Participants were selected through consecutive sampling and categorized according to KDIGO risk levels. Thyroid hormones, including free triiodothyronine (FT3), free thyroxine (FT4), and thyroid-stimulating hormone (TSH), were assessed alongside renal and metabolic markers such as estimated glomerular filtration rate (eGFR), albumin-to-creatinine ratio (ACR), serum creatinine, glycated hemoglobin (HbA1c), and duration of diabetes.

Results

Among the 350 DKD patients, 195 (55.7%) were euthyroid, 71 (20.3%) had overt hypothyroidism, 64 (18.3%) had subclinical hypothyroidism, and 20 (5.7%) had hyperthyroidism. The prevalence of thyroid dysfunction increased with KDIGO risk, showing significant variation across groups (p = 0.005). FT3 levels declined significantly from 4.15 ± 0.52 pmol/L in the low-risk group to 3.66 ± 0.73 pmol/L in the very high-risk group (F = 13.08, p < 0.001). FT4 levels also showed a significant decrease (p = 0.003), while TSH levels increased progressively (p = 0.001). FT3 was positively correlated with eGFR (r = 0.277, p < 0.001) and negatively correlated with ACR (r = -0.185, p = 0.001) and serum creatinine (r = -0.277, p < 0.001). FT4 showed a weaker positive correlation with eGFR (r = 0.150) and inverse correlations with ACR and creatinine (p < 0.05). TSH was negatively associated with eGFR (r = -0.191, p < 0.001) and positively associated with creatinine (r = 0.191, p < 0.001).

Conclusions

This study highlights a strong association between thyroid dysfunction and the severity of DKD. As KDIGO risk increased, FT3 and FT4 levels declined while TSH levels rose. These thyroid hormone alterations were significantly correlated with key renal markers, suggesting their potential utility in monitoring renal function in DKD patients.

## Introduction

Diabetic kidney disease (DKD) is a common complication of diabetes mellitus and a leading cause of chronic kidney disease (CKD) worldwide. It affects approximately 30-40% of individuals with diabetes globally, making it a major contributor to end-stage renal disease (ESRD) [1]. In Pakistan, the prevalence of DKD among diabetic patients is estimated at 23-26%, a figure that continues to rise alongside the increasing incidence of diabetes [2]. DKD is characterized by a gradual decline in renal function, typically indicated by albuminuria and a reduced glomerular filtration rate (GFR). Its pathogenesis involves a complex interplay of hyperglycemia-induced metabolic disturbances, oxidative stress, inflammation, and fibrosis, ultimately leading to endothelial dysfunction and glomerular damage [3,4].

While lifestyle changes and optimal control of blood glucose and blood pressure remain central to DKD management, recent evidence points to a possible role of thyroid dysfunction, especially hypothyroidism, in accelerating kidney damage in these patients. Managing thyroid dysfunction in DKD involves normalizing thyroid hormone levels, which may contribute to improved renal outcomes [5]. A study in patients with diabetes mellitus reported that lower levels of free triiodothyronine (FT3) and free thyroxine (FT4) were associated with poorer kidney function, whereas higher levels of thyroid-stimulating hormone (TSH) correlated with increased albuminuria and reduced renal function. Of all thyroid hormones, FT3 demonstrated the strongest association with elevated KDIGO risk, suggesting its potential involvement in the progression of DKD. Although subclinical hypothyroidism was significantly more prevalent among DKD patients, overt hypothyroidism remained relatively rare [6].

Despite the well-established link between diabetes and kidney disease, the specific relationship between thyroid hormone levels and the severity of DKD, particularly as classified by the Kidney Disease: Improving Global Outcomes (KDIGO) guidelines, remains insufficiently explored. Although some studies have addressed this connection, there is still no clear consensus in the literature [7,8]. This study was thus designed to address this gap by investigating the association between thyroid hormone profiles and DKD severity according to KDIGO risk categories. Understanding this relationship is important, as thyroid dysfunction represents a modifiable factor that, if properly managed, could potentially help improve renal outcomes in diabetic patients.

## Materials and methods

This analytical cross-sectional study was conducted at the nephrology outpatient clinic of Lahore General Hospital over a six-month period, from December 2023 to June 2024. Prior to data collection, approval was obtained from the institutional review board, and written informed consent was secured from all participants. Patient confidentiality was maintained throughout the research process.

A total of 350 patients were enrolled using a non-probability consecutive sampling technique. The sample size was calculated based on a correlation coefficient (r = 0.165) between FT4 levels and estimated GFR (eGFR), with an alpha level of 0.05 and a beta level of 0.10, resulting in a required sample size of 350 participants [6].

Inclusion criteria were as follows: patients aged 18-80 years, diagnosed with DKD for at least three months. DKD was defined as an eGFR <60 mL/min/1.73 m² for a minimum of three months - estimated using the CKD-EPI equation - and/or persistent albuminuria (urine albumin-to-creatinine ratio (ACR) ≥30 mg/g in at least two out of three consecutive urine samples). Additionally, patients with diabetes for at least five years were included if they had coexisting diabetic retinopathy and no hypoalbuminemia. This criterion specifically applied to individuals with ACR <30 mg/g and eGFR >60 mL/min/1.73 m² to capture cases in the early stages of DKD based on microvascular involvement.

Patients were excluded if they had acute kidney injury, were already diagnosed with thyroid disorders, had ESRD requiring dialysis, or had non-diabetic kidney conditions (e.g., glomerulonephritis and renal stones). Other exclusion criteria included previous or current thyroid treatments, medications that could alter thyroid function, severe systemic illnesses (e.g., heart failure and liver cirrhosis), and current or planned pregnancy.

Data collected included demographic and clinical variables such as age, gender, BMI, and duration of diabetes. Hypertension (HTN) was defined as a systolic/diastolic blood pressure ≥140/90 mmHg or current use of antihypertensive medications. Dyslipidemia was identified by elevated cholesterol or triglyceride levels or the use of lipid-lowering therapies. Renal function parameters included eGFR (CKD-EPI), serum creatinine, urinary ACR, and glycated hemoglobin (HbA1c) as a measure of glycemic control.

Thyroid function was assessed by measuring serum levels of FT3, FT4, and TSH. Based on hormonal profiles, patients were categorized as euthyroid, subclinically hypothyroid, overtly hypothyroid, or hyperthyroid. Participants were then stratified into KDIGO risk categories using combined eGFR and ACR values.

Statistical analyses were carried out using IBM SPSS Statistics for Windows, Version 26.0 (Released 2019; IBM Corp., Armonk, NY, USA). Quantitative variables were summarized as means and standard deviations, while categorical data were presented as frequencies and percentages. The normality of data distribution was assessed using the Kolmogorov-Smirnov and Shapiro-Wilk tests. Comparisons of thyroid hormone levels across KDIGO risk groups, CKD stages, and ACR categories were conducted using one-way ANOVA with Tukey’s post hoc test. Pearson correlation analysis was applied to assess relationships between thyroid hormone levels and renal parameters, including eGFR, ACR, serum creatinine, duration of diabetes, and HbA1c. Associations between categorical variables, such as thyroid dysfunction patterns and KDIGO risk groups, were examined using the chi-square test. A p-value ≤ 0.05 was considered statistically significant.

## Results

A total of 350 patients with DKD were included in the study. Of these, 178 (50.9%) were male and 172 (49.1%) were female. HTN was present in 181 patients (51.7%), while 169 patients (48.3%) had no history of HTN. Dyslipidemia was identified in 134 patients (38.3%), whereas 216 (61.7%) had no dyslipidemia.

Based on ACR categories, 115 patients (32.9%) fell into A1, 147 (42.0%) into A2, and 88 (25.1%) into A3. According to CKD staging, 29 patients (8.3%) were in stage I, 63 (18.0%) in stage II, 70 (20.0%) in stage IIIa, 53 (15.1%) in stage IIIb, 85 (24.3%) in stage IV, and 50 (14.3%) in stage V.

KDIGO risk stratification classified the cohort as follows: 39 patients (11.1%) were categorized as low risk, 65 (18.6%) as moderate risk, 62 (17.7%) as high risk, and 184 (52.6%) as very high risk.

In terms of thyroid function, 195 patients (55.7%) were euthyroid, 71 (20.3%) had overt hypothyroidism, 64 (18.3%) had subclinical hypothyroidism, and 20 (5.7%) had hyperthyroidism.

When analyzed by the KDIGO risk group, age distribution showed that most low-risk patients were between 61 and 80 years (64.1%), while very high-risk patients were predominantly aged 41-60 years (48.4%) and 61-80 years (40.8%), indicating a significant association (p = 0.002). HTN was significantly more prevalent in the very high-risk group (57.6%) compared to the low-risk group (28.2%) (p = 0.008). Both the ACR category and CKD stage showed strong associations with KDIGO risk levels (p < 0.001 for both). However, no significant associations were observed for sex (p = 0.560) or dyslipidemia (p = 0.149).

Overall, increasing KDIGO risk was associated with advancing CKD stages and higher ACR levels (Table 1).

**Table 1:** Comparison of clinical and laboratory characteristics across KDIGO risk categories (n = 350) Comparison of clinical, biochemical, and thyroid function parameters across KDIGO risk categories in DKD patients. Continuous variables (age, BMI, eGFR, serum creatinine, ACR, HbA1c, and diabetes duration) were analyzed using one-way ANOVA, while categorical variables (age group, sex, HTN, dyslipidemia, ACR category, CKD stage, and thyroid dysfunction) were assessed via Pearson’s chi-square test. Statistical significance was set at p ≤ 0.05. ACR, albumin-to-creatinine ratio; CKD, chronic kidney disease; DKD, diabetic kidney disease; eGFR, estimated glomerular filtration rate; HbA1c, glycated hemoglobin; HTN, hypertension; KDIGO, Kidney Disease: Improving Global Outcomes

Variable	Low risk (n = 39)	Moderate risk (n = 65)	High risk (n = 62)	Very high risk (n = 184)	Test value	p-Value	95% CI
Age (years)	60.97 ± 15.15	53.02 ± 14.96	56.18 ± 15.15	55.90 ± 12.26	F = 2.766	0.042	54.11-57.68
18-40 years	7 (17.9%)	17 (26.2%)	12 (19.4%)	20 (10.9%)	χ² = 20.66	0.002	-
41-60 years	7 (17.9%)	22 (33.8%)	23 (37.1%)	89 (48.4%)			
61-80 years	25 (64.1%)	26 (40.0%)	27 (43.5%)	75 (40.8%)			
Male	16 (41.0%)	36 (55.4%)	32 (51.6%)	94 (51.1%)	χ² = 2.059	0.56	-
Female	23 (59.0%)	29 (44.6%)	30 (48.4%)	90 (48.9%)			
BMI (kg/m²)	27.69 ± 2.05	26.46 ± 2.01	26.33 ± 2.34	25.33 ± 2.82	F = 11.148	<0.001	24.92-25.74
eGFR (mL/min/1.73m²)	87.39 ± 14.24	70.00 ± 18.75	56.96 ± 19.61	24.61 ± 12.01	F = 282.202	<0.001	22.66-26.36
Serum creatinine (mg/dL)	0.81 ± 0.20	1.08 ± 0.35	1.33 ± 0.41	2.97 ± 1.28	F = 111.021	<0.001	2.78-3.15
ACR (mg/g)	19.51 ± 5.94	97.72 ± 95.98	233.08 ± 241.34	301.38 ± 250.57	F = 27.803	<0.001	264.93-337.83
A1 (<30 mg/g)	39 (100%)	28 (43.1%)	17 (27.4%)	31 (16.8%)	χ² = 131.306	<0.001	-
A2 (30-300 mg/g)	0 (0.0%)	37 (56.9%)	29 (46.8%)	81 (44.0%)			
A3 (>300 mg/g)	0 (0.0%)	0 (0.0%)	16 (25.8%)	72 (39.1%)			
Diabetes duration (years)	7.08 ± 3.58	7.82 ± 3.56	8.31 ± 3.37	9.14 ± 3.16	F = 5.650	0.001	8.68-9.60
HbA1c (%)	7.57 ± 0.58	7.97 ± 0.74	8.25 ± 1.13	8.05 ± 1.46	F = 2.560	0.055	7.84-8.26
HTN	11 (28.2%)	35 (53.8%)	29 (46.8%)	106 (57.6%)	χ² = 11.916	0.008	-
Dyslipidemia	9 (23.1%)	25 (38.5%)	22 (35.5%)	78 (42.4%)	χ² = 5.337	0.149	-
CKD Stage 1	16 (41.0%)	9 (13.8%)	4 (6.5%)	0 (0.0%)	χ² = 369.456	<0.001	-
CKD Stage II	23 (59.0%)	28 (43.1%)	12 (19.4%)	0 (0.0%)			
CKD Stage IIIa	0 (0.0%)	28 (43.1%)	29 (46.8%)	13 (7.1%)			
CKD Stage IIIb							
0 (0.0%)	0 (0.0%)	17 (27.4%)	36 (19.6%)				
CKD Stage IV	0 (0.0%)	0 (0.0%)	0 (0.0%)	85 (46.2%)			
CKD Stage V	0 (0.0%)	0 (0.0%)	0 (0.0%)	50 (27.2%)			
Euthyroidism	29 (74.4%)	46 (70.8%)	38 (61.3%)	82 (44.6%)	χ² = 23.671	0.005	-
Hypothyroidism	3 (7.7%)	7 (10.8%)	10 (16.1%)	51 (27.7%)			
Subclinical hypothyroidism	5 (12.8%)	9 (13.8%)	11 (17.7%)	39 (21.2%)			
Hyperthyroidism	2 (5.1%)	3 (4.6%)	3 (4.8%)	12 (6.5%)			

FT3 levels showed a progressive decline with increasing KDIGO risk severity (F = 13.08, p < 0.001), with values decreasing from 4.15 ± 0.52 pmol/L in low-risk patients to 3.66 ± 0.73 pmol/L in very high-risk patients. FT4 was significantly lower in the very high-risk group (16.11 ± 1.16 pmol/L) compared to the low-risk group (16.69 ± 0.77 pmol/L; F = 4.64, p = 0.003). TSH levels increased with higher KDIGO risk (F = 5.81, p = 0.001), reaching a peak of 4.04 ± 2.46 mIU/L in very high-risk patients compared to 2.76 ± 1.94 mIU/L in low-risk patients (Table 2).

**Table 2:** Comparison of thyroid hormone levels across KDIGO risk categories in DKD patients (n = 350) One-way ANOVA was used to assess statistical differences in thyroid hormone levels across KDIGO risk categories (df = 3,346). A p-value ≤ 0.05 was considered statistically significant. DKD, diabetic kidney disease; FT3, free triiodothyronine; FT4, free thyroxine; KDIGO, Kidney Disease: Improving Global Outcomes; TSH, thyroid-stimulating hormone

Variables	Low risk (n = 39)	Moderate risk (n = 65)	High risk (n = 62)	Very high risk (n = 184)	t-Test value (F)	p-Value	95% CI (lower-upper)
FT3 (pmol/L)	4.15 ± 0.52	4.15 ± 0.52	3.77 ± 0.52	3.66 ± 0.73	13.08	<0.001	3.75-3.89
FT4 (pmol/L)	16.69 ± 0.77	16.51 ± 0.81	16.29 ± 1.08	16.11 ± 1.16	4.64	0.003	16.17-16.40
TSH (mIU/L)	2.76 ± 1.94	3.01 ± 1.92	3.40 ± 2.12	4.04 ± 2.46	5.81	0.001	3.35-3.84

The correlation analysis revealed a significant positive correlation between FT3 and eGFR (r = 0.277, p < 0.001), while FT3 showed a negative correlation with ACR (r = -0.185, p = 0.001) and serum creatinine (r = -0.277, p < 0.001). Similarly, FT4 demonstrated a weak but significant positive correlation with eGFR (r = 0.150, p = 0.005) and a negative correlation with ACR (r = -0.112, p = 0.036) and serum creatinine (r = -0.150, p = 0.005). TSH was negatively correlated with eGFR (r = -0.191, p < 0.001) and positively correlated with serum creatinine (r = 0.191, p < 0.001). However, there were no statistically significant correlations between thyroid hormones and HbA1c or diabetes duration (p > 0.05) (Table 3).

**Table 3:** Correlation of thyroid hormones (FT3, FT4, and TSH) with renal and metabolic markers ACR, albumin-to-creatinine ratio; eGFR, estimated glomerular filtration rate; FT3, free triiodothyronine; FT4, free thyroxine; HbA1c, glycated hemoglobin; TSH, thyroid-stimulating hormone

Variables	FT3 (r, p-Value)	FT4 (r, p-Value)	TSH (r, p-Value)
eGFR	0.277, p < 0.001	0.150, p = 0.005	-0.191, p < 0.001
ACR	-0.185, p = 0.001	-0.112, p = 0.036	0.082, p = 0.126
HbA1c	0.006, p = 0.905	-0.005, p = 0.925	0.019, p = 0.729
Diabetes duration	-0.060, p = 0.265	-0.006, p = 0.913	-0.007, p = 0.898
Serum creatinine	-0.277, p < 0.001	-0.150, p = 0.005	0.191, p < 0.001

**Figure 1:**
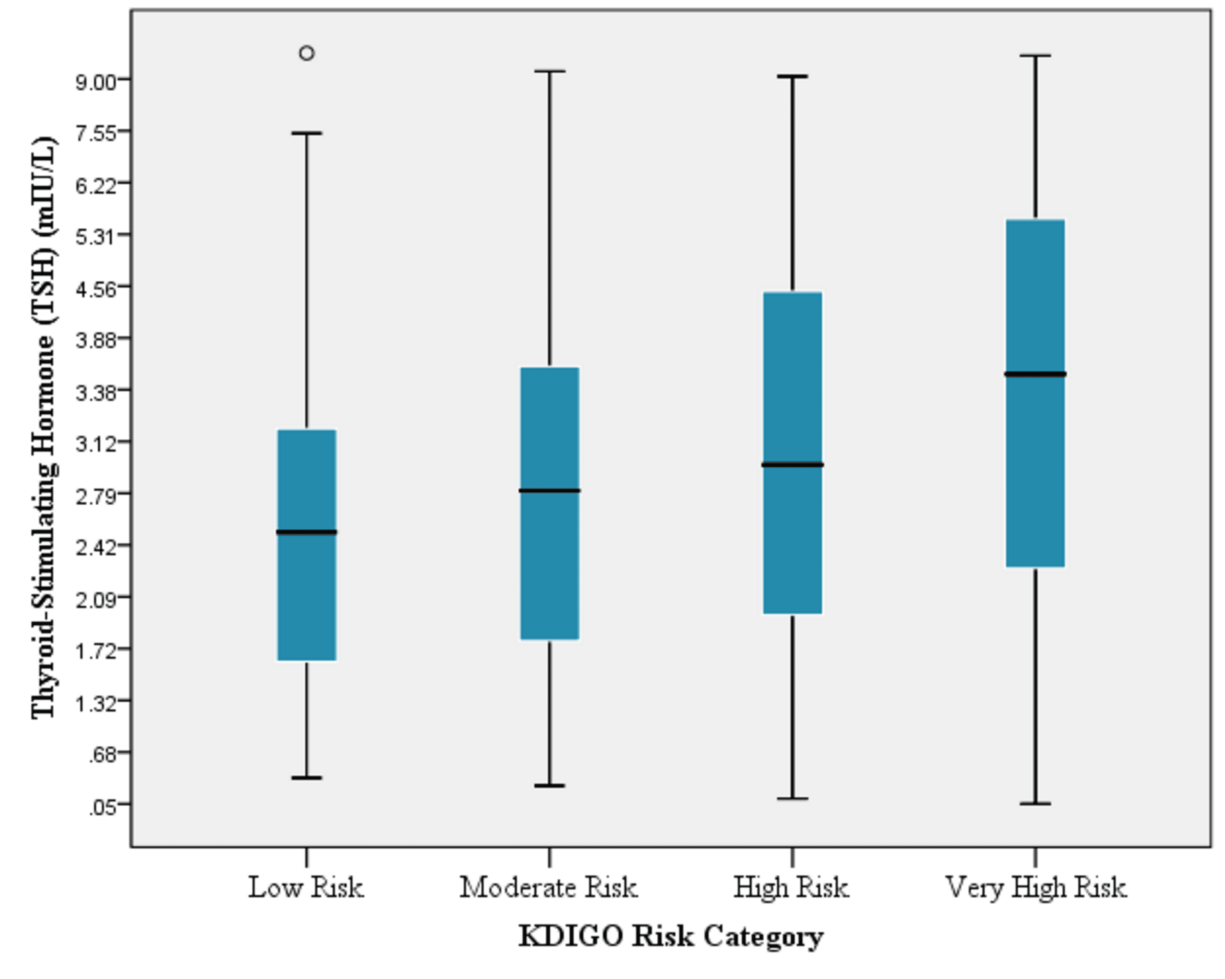
Box plot of TSH levels across KDIGO risk categories KDIGO, Kidney Disease: Improving Global Outcomes; TSH, thyroid-stimulating hormone

The box plot shows progressively higher median TSH levels across increasing KDIGO risk categories, with the highest values observed in the very high-risk group (p = 0.001) (Figure 1).

## Discussion

In this study, the baseline characteristics of 350 DKD patients revealed distinct trends across KDIGO risk categories. Surprisingly, our low-risk patients were older (60.97 ± 15.15 years) than moderate-risk patients (53.02 ± 14.96 years, p=.042). This contradicts what most studies show. Pan et al. (2019) found age increased with CKD severity (52.7 years in CKD1 to 70.5 years in CKD5) [9]. Liu et al. (2023) similarly reported older patients in higher KDIGO risk groups. Our different finding might be because we included both type 1 and type 2 diabetes patients, with type 1 patients often developing CKD at younger ages [7]. Shi et al. (2023) found a mean age of 61.64 years in type 2 patients and a 10-year diabetes duration, similar to our very high-risk group (9.14 ± 3.16 years) [10]. While age is often considered a predictor of CKD progression, the findings of this study suggest that KDIGO risk in DKD patients may depend more on factors such as diabetes type, glycemic control, and associated comorbidities, rather than age alone. In this study, diabetes duration increased with KDIGO risk, peaking in the very high-risk group (9.14 ± 3.16 years, p = 0.001). This aligns with Zou et al. (2018), who also found longer diabetes duration in DKD patients than in non-DKD [11]. Zhang et al. (2018) and Liu et al. (2023) reported similar findings, supporting a strong link between longer diabetes history and worsening kidney status [7,12]. BMI reported a decline with the rising KDIGO risk category, from 27.69 ± 2.05 kg/m² in low-risk to 25.33 ± 2.82 kg/m² in very high-risk patients (p < 0.001). Similarly, Zou et al. (2018) found lower BMI in DKD patients (25.92 ± 3.35 kg/m²) compared to non-DKD groups [11]. In contrast, Yang et al. (2022) noted slightly higher BMI in DKD patients, though not statistically significant [6]. Shi et al. (2023) found no BMI variation across renal risk [10]. These contradictions may arise from varying study populations - our findings likely highlight weight loss in severe DKD, while others reflect metabolic risks in earlier stages. This mix of results underscores the need to consider disease progression and population differences when interpreting BMI trends in DKD.

This study reported a significant decrease in renal function as KDIGO risk increased. A decreasing trend in eGFR was accompanied by a rise in serum creatinine and albuminuria, indicating progressive kidney impairment. Similar patterns have been reported by Yang et al. (2022), Liu et al. (2023), and Pan et al. (2019), supporting the association between worsening kidney function and higher KDIGO risk [6,7,9]. These consistent findings underscore the clinical utility of these markers in assessing and monitoring DKD progression. HbA1c levels showed a mild upward trend with increasing KDIGO risk but did not reach statistical significance (p = 0.055). This observation is similar to Sibanda et al. (2024), who also found no significant association between HbA1c and renal or thyroid parameters [13]. However, these findings contrast with Liu et al. (2023) and Zou et al. (2018), both of whom reported significantly higher HbA1c levels in patients with more advanced kidney involvement [7,11]. The findings suggest that factors other than glycemic control may play a more central role in DKD progression in our cohort, highlighting the need for a broader approach to risk evaluation and management.

FT3 showed a significant positive correlation with eGFR (r = 0.277, p < 0.001) and negative correlations with serum creatinine (r = -0.277, p < 0.001) and ACR (r = -0.185, p = 0.001). These findings are supported by Shi et al. (2023), who reported FT3 positively correlated with eGFRcys (r = 0.326) and eGFRcr (r = 0.324) and negatively with UACR (r = -0.255) [10]. Demir et al. (2024) found FT3 positively correlated with eGFR (r = 0.395) and negatively with ACR (r = -0.264) [14]. Similarly, Liu et al. (2023) observed stronger correlations with eGFR (r = 0.49) and ACR (r = -0.29), while Chen et al. (2020) also noted consistent FT3 associations with eGFR (r = 0.49) and UACR (r = -0.29) [7,15]. Pan et al. (2019) reported a positive FT3-eGFR correlation (r = 0.239), and Yang et al. (2022) showed FT3 positively correlated with eGFR (r = 0.325) and negatively with ACR (r = -0.267) [6,9]. These repeated observations across various populations suggest a reproducible link between FT3 and renal function markers. FT4 in this study showed a weak but significant positive correlation with eGFR (r = 0.150, p = 0.005) and inverse correlations with ACR (r = -0.112, p = 0.036) and serum creatinine (r = -0.150, p = 0.005). These findings agree with Yang et al. (2022) (eGFR: r = 0.165; ACR: r = -0.109) and Liu et al. (2023) (eGFR: r = 0.18; ACR: r = -0.11), though overall FT4 remains a weaker prognostic marker for DKD progression than FT3 [6,7]. TSH in this study was negatively correlated with eGFR (r = -0.191, p < 0.001) and positively with serum creatinine (r = 0.191, p < 0.001), indicating a link between declining renal function and rising TSH. These findings align with Liu et al. (2023), who reported similar correlations (eGFR: r = -0.20; ACR: r = 0.21, p < 0.001), and Zhang et al. (2018), who found a weaker negative correlation between TSH and eGFR (r = -0.086, p < 0.01), suggesting variable TSH sensitivity across renal stages [7,12].

In this study, hypothyroidism and subclinical hypothyroidism increased notably with rising KDIGO risk, reaching 27.7% and 21.2% in the very high-risk group, while euthyroidism declined to 44.6%. These findings align with Liu et al. (2023), Pan et al. (2019), and Sibanda et al. (2024), who all reported increased thyroid dysfunction in advanced CKD [7,9,13]. Similar trends were also noted by Yang et al. (2022), Zhang et al. (2018), and Demir et al. (2024), highlighting a likely interplay between declining renal function and thyroid dysregulation [6,12,14].

This study’s strengths include its ample sample size and detailed thyroid-renal profiling across KDIGO categories, aligning with global trends. However, its cross-sectional nature limits causal inference, and key thyroid markers like reverse T3 and antibodies were not assessed. Future studies should adopt longitudinal designs to clarify thyroid-DKD causality, integrate thyroid imaging/antibody testing, and assess antidiabetic and antiproteinuric medication effects on thyroid function and mechanisms linking thyroid dysfunction with DKD progression. Addressing these gaps could refine risk prediction and therapeutic strategies in DKD.

## Conclusions

The findings of this study indicate a clear association between thyroid hormone alterations and the progression of DKD. As KDIGO risk categories increased, FT3 and FT4 levels declined, while TSH levels rose significantly. Correlation analysis further supported this pattern, showing consistent associations of FT3 and FT4 with renal markers such as eGFR, serum creatinine, and ACR. A higher prevalence of hypothyroidism and subclinical hypothyroidism was also observed in patients with more advanced kidney disease. These results underscore the relevance of routine thyroid function assessment in DKD, suggesting its utility in risk stratification and early identification of progressive renal dysfunction.
